# Compressive Strength Testing of Glass-Fibre-Reinforced Tooth Crown Tissues After Endodontic Treatment

**DOI:** 10.1007/s10439-023-03377-w

**Published:** 2023-10-04

**Authors:** Monika Ostapiuk, Janusz Tarczydło, Katarzyna Kamińska, Barbara Surowska, Bożena Tarczydło

**Affiliations:** 1 Department of Materials Engineering, Faculty of Mechanical Engineering, Lublin University of Technology, Nadbystrzycka St. 36, 20-618 Lublin, Poland; 2 Faculty of Medicine, Dentistry, Medical University of Lublin, Chodźki St. 6, 20-093 Lublin, Poland; 3 Department of Conservative Dentistry with Endodontics, Medical University of Lublin, Chodźki St. 6, 20-950 Lublin, Poland

**Keywords:** Compression test, Root-canals, EverX Posterior, Hahnenkratt, Micro-CT

## Abstract

The objective of this study was to compare the effects of using short and continuous fibres for repairing compression-induced tooth crown damage. Human teeth were used for the study. They were upper medial incisors and maxillary first premolars lost due to periodontal causes. The teeth were divided into two groups with Hahnenkratt and short glass fibres. Teeth compressive strength tests were carried out. Then micro-CT imaging of the teeth and their fractures obtained after compression was performed. The teeth restored with Hahnenkratt’s glass fibre posts showed higher compressive strength than the teeth restored using the EverX Posterior material. The tooth’s most weakened and sensitive point after endodontic treatment was the cervical area of the tooth. All cracks were parallel to the root canal.

## Introduction

The development of dentistry and the growing requirements of biomaterials stimulate the search for materials with high physical, chemical and mechanical properties [1]. Restorative dentistry emphasises the preservation of dental structures. An ideal dental restoration should present perfect marginal adaptation, biocompatibility with the oral environment and aesthetics [2–4]. The reconstruction of tooth tissues after root canal treatment is a procedure for restoring the function and aesthetics of the treated tooth. The choice of material, the type of restoration and its accuracy are fundamental, as studies show that the main cause of teeth loss after endodontics is improper restoration. A study by Vir [5] demonstrated that a common cause of extraction for root-canal-treated teeth is their fracture and breakage due to incorrect restoration. Similar observations were made by Sjogren et al. [6], who found that 11% of endodontically treated teeth were extracted due to incorrect restoration. On the other hand, Sorensen et al. [7] showed that poor restoration could lead to periapical tissue lesions due to bacterial microleakage and reinfection in the endodontic system [8]. Variations in mineral properties in bones and teeth contribute to the mechanical performance of these tissues [9]. The weakening of an endodontically treated tooth depends on the tooth tissue loss due to both the pathological process and the treatment itself. According to studies conducted by endodontists [10–12], root canal treatment leads to a significant weakening of the tooth structure not only due to tissue loss, but also due to dentin dehydration, loss of blood circulation in the pulp and changes in collagen. Importantly, root canal treatment significantly weakens the tooth structure not only due to tissue loss, but also due to dentine dehydration, loss of circulation in the pulp or changes in collagen. In addition, chemical compounds used in root canal treatment can also affect the mechanical strength of mineralised tissues [13].

Endodontically treated teeth with several structural defects often require a post to maintain crown restoration. Compared to metal posts, fibre-reinforced resin posts are a better solution for endodontically treated teeth [14, 15].

One of the key functions of fibres in a composite material is to translocate stresses and ensure adequate material stiffness. The strength properties of reinforcing fibres directly impact the strength properties of the composite material. The fibres are placed such that its (composite material) longitudinal axis is aligned perpendicular to the compressive forces to increase the strength of restoration. However, if the longitudinal axis of fibre is parallel to compressive forces, no enhancement will occur in restoration [16–18].

This study aimed to compare the effects of short fibres (EverX Posterior) and continuous fibres (glass fibre posts Hahnenkratt, Germany) for repairing compression-induced tooth crown damage.

## Materials and Methods

### Materials

Human teeth were used for the study. They were upper medial incisors and maxillary first premolars lost due to periodontal causes. The consent of the bioethics committee was number KE-0254/339/2016. The teeth were stored in saline for 24 h after removal. Teeth for endodontic treatment were caries free and without fillings. In compliance with the tenets of minimally invasive dentistry, the following was performed after gaining access to the tooth cavity: the pulp chamber roof was removed and its contents removed; the chamber and canal orifices were prepared and their contents were removed; the working length was determined; the root canals were prepared by the traditional method using the ProTaper Next rotary instrumentation (DentSply, Sirona). The manufacturer's recommended tool sequence was employed. The root canal preparation was completed with a 35-size file. 2% NaOCl was used as a rinse agent during mechanical preparation. The canals were rinsed with 0.9% NaOCl upon canal preparation completion and dried with absorbent points. The canals were then filled by lateral condensation technique using AH Plus sealant.

The teeth were divided into two groups: 10 teeth each (5 incisors and 5 maxillary first premolars). In Group 1, short glass-fibre-reinforced composite EverX Posterior (GC, Japan) was used for crown reinforcement—this material was coated with a layer of conventional composite CeramX (DentsPly, USA). The incisors teeth from Group 1 will be named in the text in addition to premolar teeth. Posterior is composed of randomly oriented short E-glass fibre and inorganic barium glass particles fillers in combination with a semi-interpenetrating polymer network matrix that consists of bisphenol A glysidyl methacrylate, triethylene glycol dimethacrylate, and polymethylmethacrylate. The glass fibres are pre-impregnated into the composite resin and are 0.8 mm in average length.

In Group 2, continuous fibres (glass fibre posts Hahnenkratt, Germany) were used for tooth crown reinforcement. The incisors teeth from Group 2 will be named in the text in addition to premolar teeth. The glass fibre posts were cemented after removing gutta-percha from the canals to a depth of 5 mm and cleansing the canal walls of a smear layer. The GC G-aenial Bond system and Dual Luting cement (BuildIt, USA) were used for post-cementation. After that, a filling made of CeramX composite material (DentsPly, USA) was inserted. Details of the materials are given in Table 1.

**Table 1:** The products used for tooth restorations in Groups 1 and 2

Product	Type/composition	Manufacturer
Build-It FR	Self-adhesive luting agent Bis-GMA, UDMA, HDDMA, barium borosilicate glass fillers, chopped glass fibre, photochemical initiator	Pentron Clinical Technologies, USA
Group 2 Hahnenkratt, posts	Cytec Blanco: Glass fibre, epoxy resin matrix	Hahnenkratt, Konigsbach-Stein, Germany
G-aenial bond	Self-etch adhesive, 4-MET, 10-MDP, Glycerol dimethacrylate, TEGDMA, water, acetone, initiators	GC Corporation, Tokyo, Japan
Group 1 EverX Posterior	Fibre-reinforced composite Resin: semi-INP: net-PMMA inter-net-poly (Bis-GMA): Bis-GMA, TEGDMA, PMMA Fillers: E-glass fibre, barium borosilicate (57% vol.)	GC Corporation, Tokyo, Japan
Group 1 and Group 2 Ceram.X SphereTEC^TM^one	UDMA, Bis-EMA and TEGDMA Spherical prepolymerised filler, non-agglomerated barium glass and ytterbium fluoride and colloidal silica with a mean particle size of 0.8 μm 59–61% vol.	Dentsply/Caulk, Milford, DE, USA

To ensure optimum stress distribution during compression testing, the tooth cusp was reduced to obtain a uniform and flat tooth surface.

### Micro-CT Examination

To investigate the problem of using Group 1 and Group 2 for the restoration of teeth after endodontic treatment, a micro-CT analysis of tooth structure quality was conducted. The teeth were examined using a SkyScan 1174 micro-CT scanner (Bruker, Belgium) with a 1.3MP (1024 × 1024) VDS camera. Scanning parameters and software used for image analysis are given in Table 2. The internal structure of the teeth was examined both before and after the compression tests.

**Table 2:** Scanning parameters and software used for image analysis

Scanning parameters	
X-ray source	50 kV
Tube current	520 μA
Image resolution	15.13 μm
Exposure time	3400 ms
Rotation angle	0.7°
Average frame rate	3
Filter	Al. 0.5
Scanning time	1 h 6 min
Software	
For image reconstruction: NRecon Ver. 1.6.10.4	
For 2D image analysis: Data Viewer Ver.1.5.2.4	
For 3D image analysis: CTVox Ver. 3.1.2	

### Compressive Strength Testing

Compressive strength tests were conducted on a static testing machine (SHIMADZU, Japan) with a load range of 20 kN according to ISO 9917-1: 2007 [19]. The mechanical test compression precision was with a maximum of ± 1% of the indicated value. The tests were performed at a feed rate of 0.5 mm/min. This was given by a stainless-steel bar with a hemispherical head, which was attached to the upper movable compartment. The testing parameters were room temperature of 22 °C ± 1 °C and a preload force of 0.1 N (Newton), recorded by a computer. This machine records—with accuracy—any minimal force changes applied to the samples. When the restorations were fractured, the compressive test was considered completed. The teeth were placed in a special holder with a custom-fitted shape and imitating the gums placed on the tooth. The tooth was immobilised as it is in the jaw, and a compressive force acted on it from above.

Compression strength was determined based on the formula in Eq. 1. The tests were continued until tooth failure.

1 $$\sigma =\frac{P}{A},$$

*σ* compressive stress, *P* load or force, *A* cross-sectional area of a specimen.

### Fracture Analysis

Fractured surfaces after compression testing were examined using the NIKON SMZ 1500 stereo microscope. In addition, filling damage was examined by CT.

## Results and Discussion

### Micro-CT Examination After Image Reconstruction

Figures 2, 3, 4, and 5 show reconstructed 2D and 3D images in the XYZ axis, which were used to examine the internal structure of the fillings and teeth.

Figures 2 and 3 show the X-ray images of single- and double-rooted teeth restored with EverX Posterior and CeramX Sphere Tec (Group 1).

**Fig. 1:**
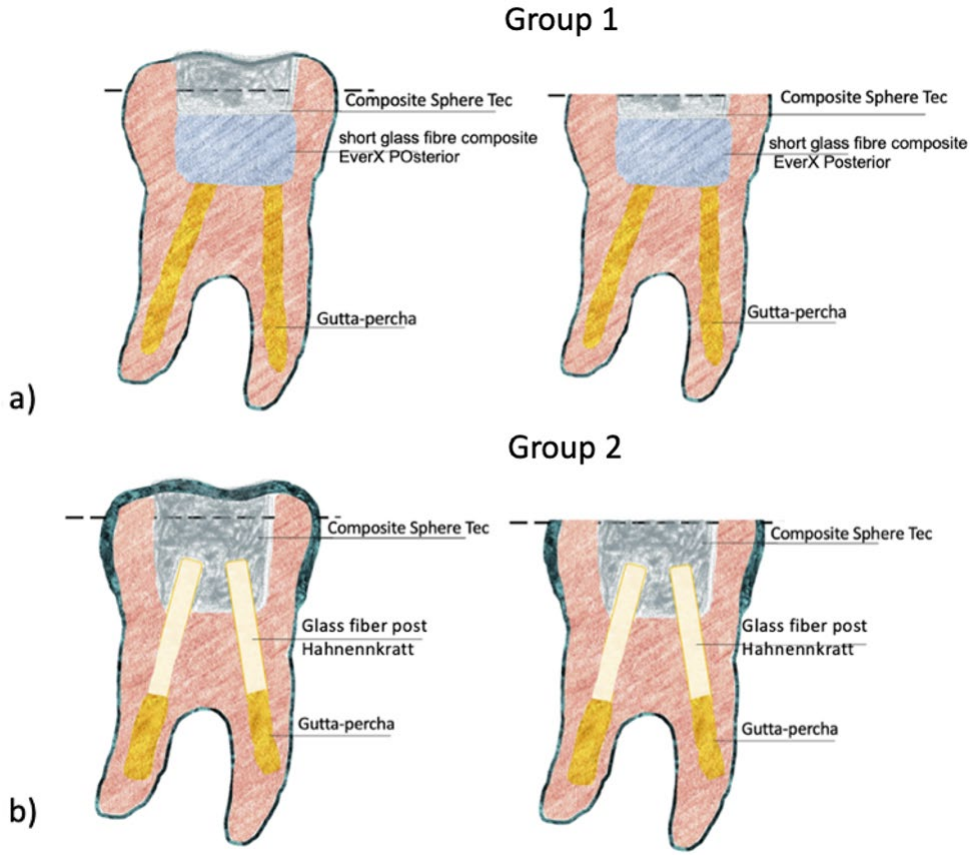
View of Group 1 and 2 teeth with **a** glass fibre posts Hahnenkratt; **b** uniform upper surface; **c** short fibres EverX Posterior; **d** uniform upper surface.

**Fig. 2:**
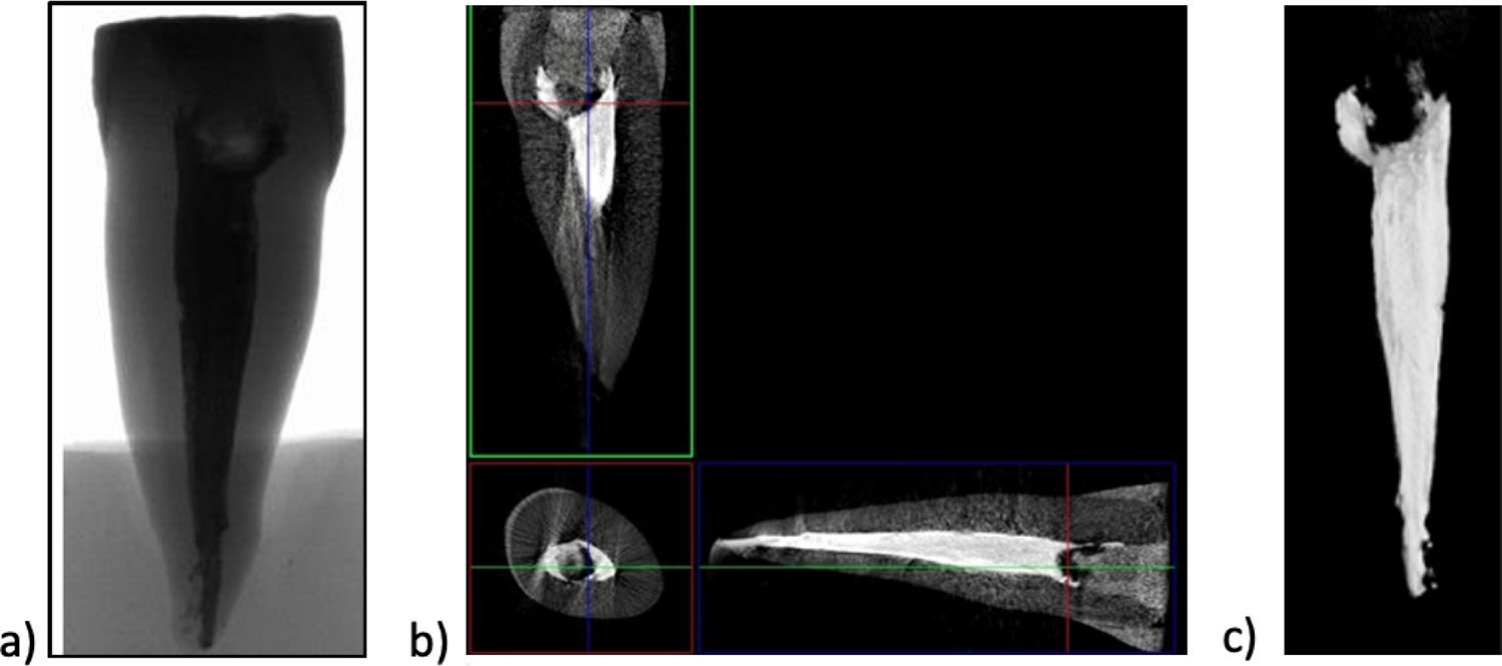
X-ray images of an upper incisor tooth **a** 2D; **b** 3D; **c** tooth filled with short glass-fibre-reinforced composite EverX Posterior material; micro-CT

**Fig. 3:**
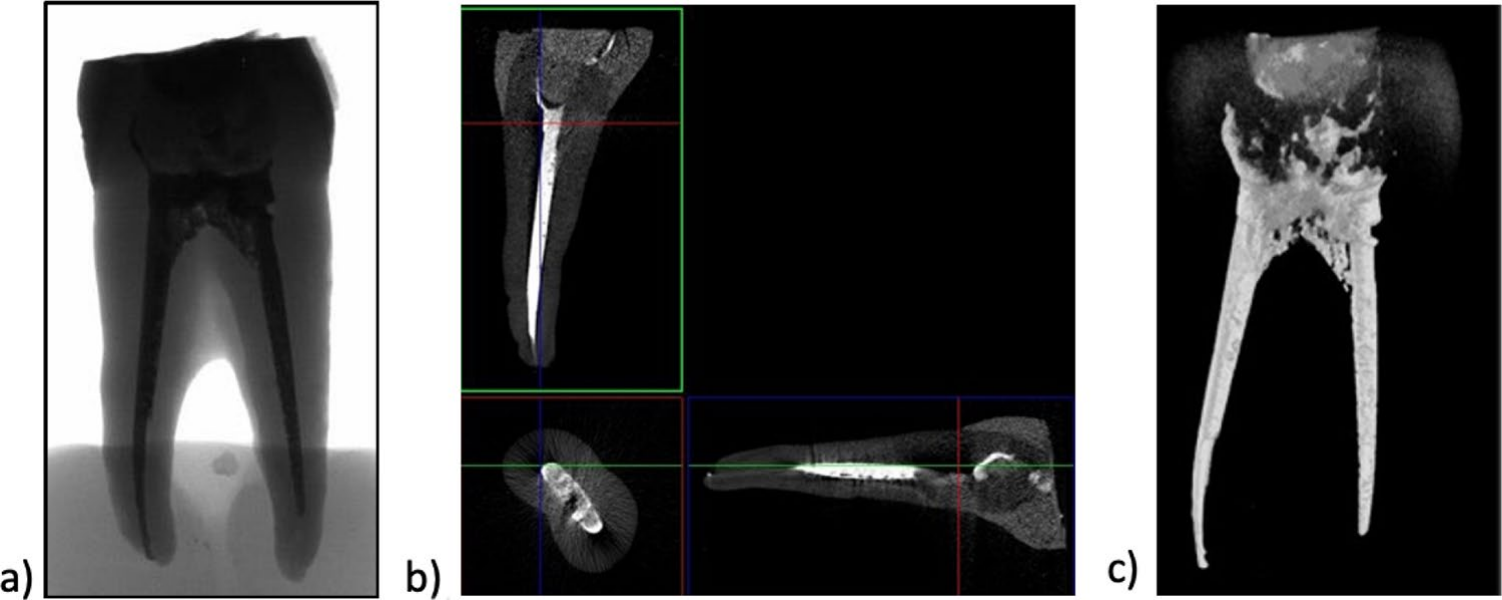
X-ray images of an upper incisor and premolar tooth **a** 2D; **b** 3D; **c** tooth filled with short glass-fibre-reinforced composite EverX Posterior material; micro-CT

The X-ray image (Fig. 2a) of the incisal reveals a homogeneous structure as well as complete filling of the tooth (Fig. 1). The reconstructed image in the XYZ axis (Fig. 2b) shows individual tissues of the tooth, root canal obturation and tooth crown cavity filling. Figure 3c shows the 3D image of an entire single-canal tooth reinforced after endodontic treatment with EverX Posterior and CeramX Sphere Tec. The short-fibre-reinforced composite EverX Posterior material filling shows no visible porosities or discontinuities in its structure. No discontinuity is observed between the tooth tissue and the composite filling. The areas marked with an intermediate shade of grey are due to the bonding system.

An analysis of the premolar tooth filled with EverX Posterior (Fig. 3) reveals a discontinuity at the filling/tooth tissue interface (especially in the neck area) as well as root canal underfill (Fig. 3b). No defects in the form of porosity can be observed inside the filling in the 2D image. The fibres in the EverX Posterior are invisible in the CT image due to similar X-ray absorption coefficients of the glass fibre and the composite matrix.

The 3D image of the filling (Fig. 3c) shows the tooth structure with a filling. The tooth canal filling is uniform (with no discontinuities) and the visible darker area is the bonding system.

**Fig. 4:**
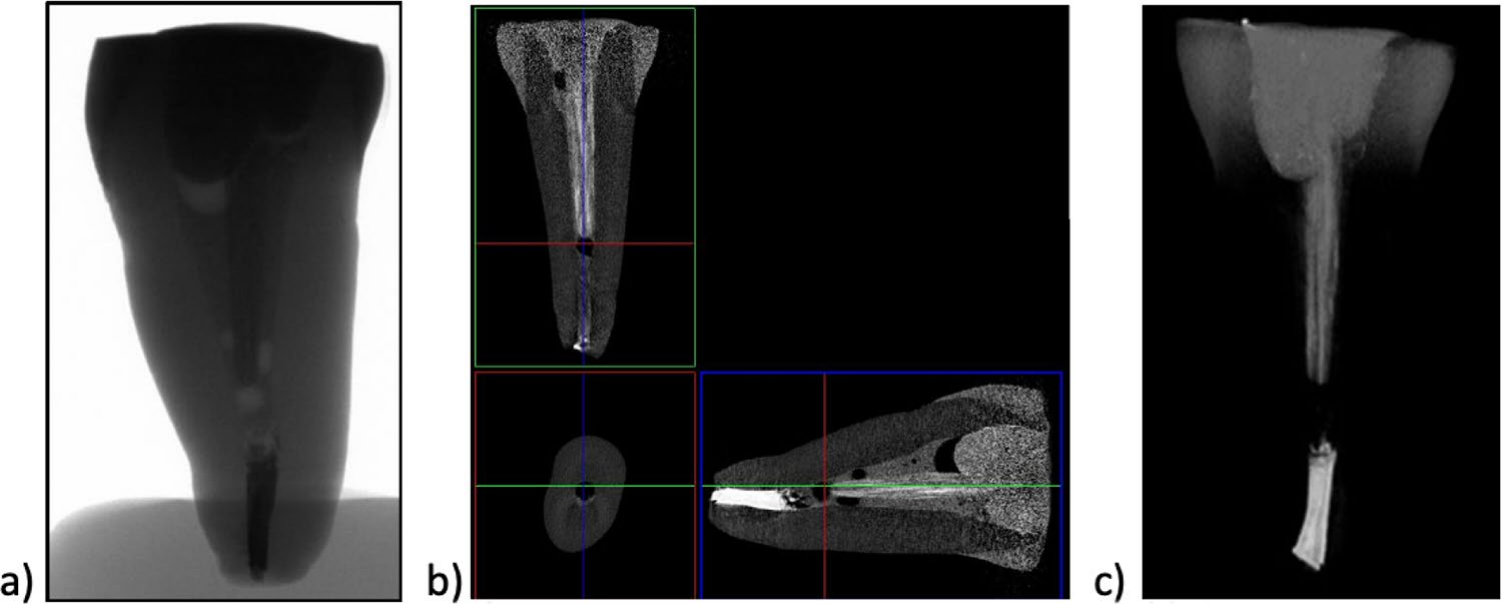
X-ray images of an incisor: **a** 2D; **b** 3D; **c** tooth with continuous fibres glass posts Hahnenkratt; micro-CT

**Fig. 5:**
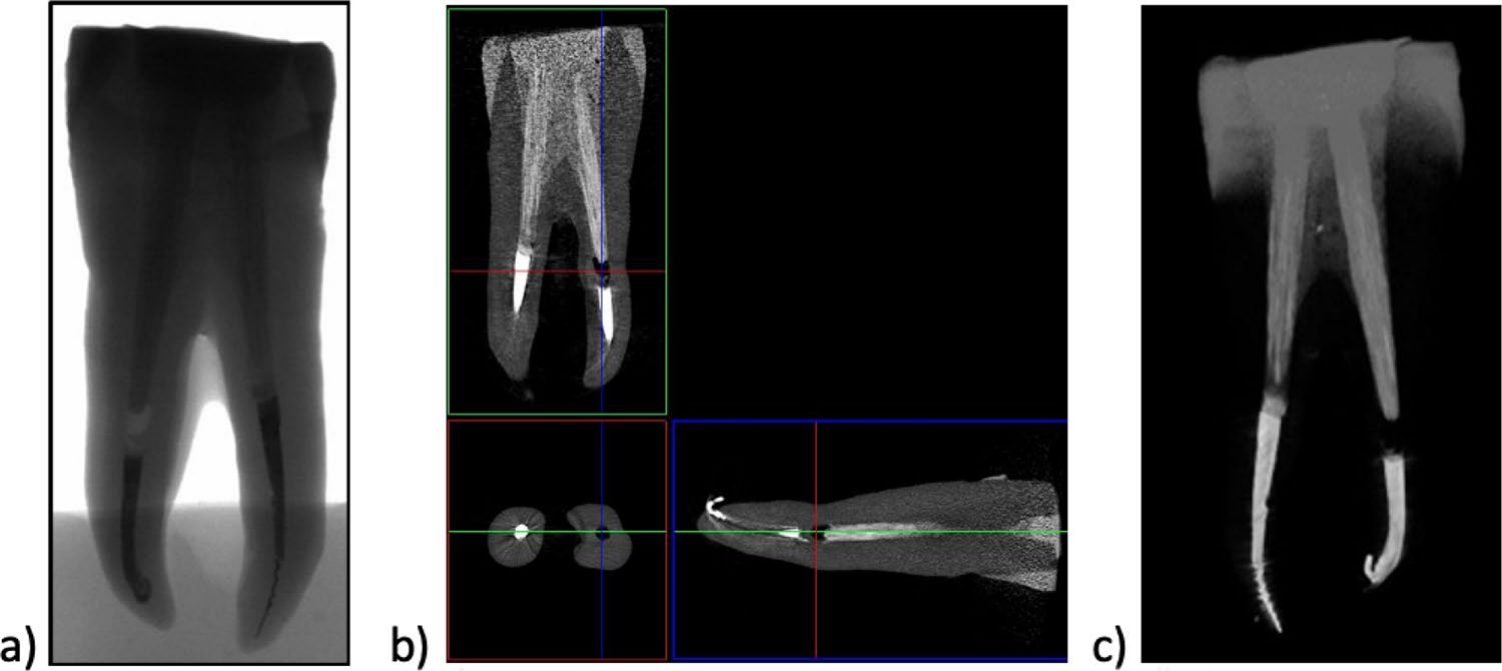
X-ray images of a premolar tooth: **a** 2D; **b** 3D; **c** continuous fibres glass posts Hahnenkratt; micro-CT

The teeth shown in Figs. 4 and 5 were reconstructed using continuous fibres glass posts Hahnenkratt. As mentioned previously, one can observe the presence of dentin, enamel, root-crown post, gutta-percha, filling and bonding material, which results from differences in radiation absorption (Group 2). The premolar tooth (Fig. 4b) shows a discontinuity between the root canal filling and the glass fibre post, which can be explained by the presence of the bonding system used in this region. The glass fibre post is shown in the images reconstructed in the *XYZ* axis. The arrangement of the continuous fibres in the tooth crown is clearly visible (Fig. 4b and b).

**Fig. 6:**
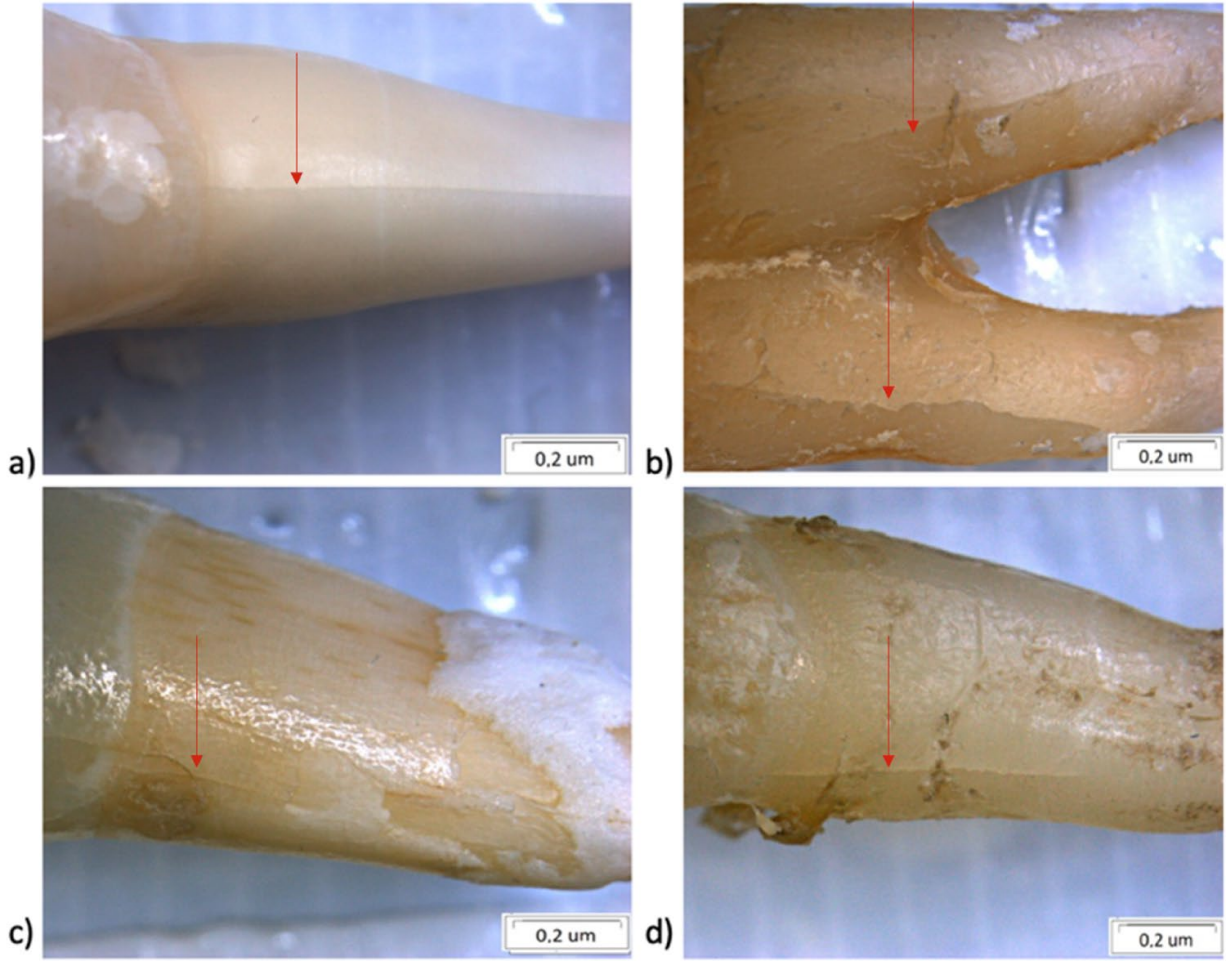
Photographs of tested teeth: **a** incisor teeth with short glass-fibre-reinforced composite EverX Posterior; **b** premolar teeth with short glass-fibre-reinforced composite EverX Posterior; **c** incisor teeth with continuous fibres glass posts Hahnenkratt; **d** premolar teeth with continuous fibres glass posts Hahnenkratt—view of a single root; stereo microscope

Figure 6 shows teeth after compression testing. All the teeth have developed a crack along their roots. In addition, one can observe enamel chipping (Fig. 6a) and cracking in the lower part of the tooth crown (Fig. 6c). Since no changes were observed in the crown region of the premolar teeth, Fig. 6b and d only shows the root part with cracks.

### Micro-CT Examination After Compressive Strength Testing

To observe internal changes induced after the compressive strength test, the structure was re-examined by micro-CT, and 2D/3D images were captured again.

**Fig. 7:**
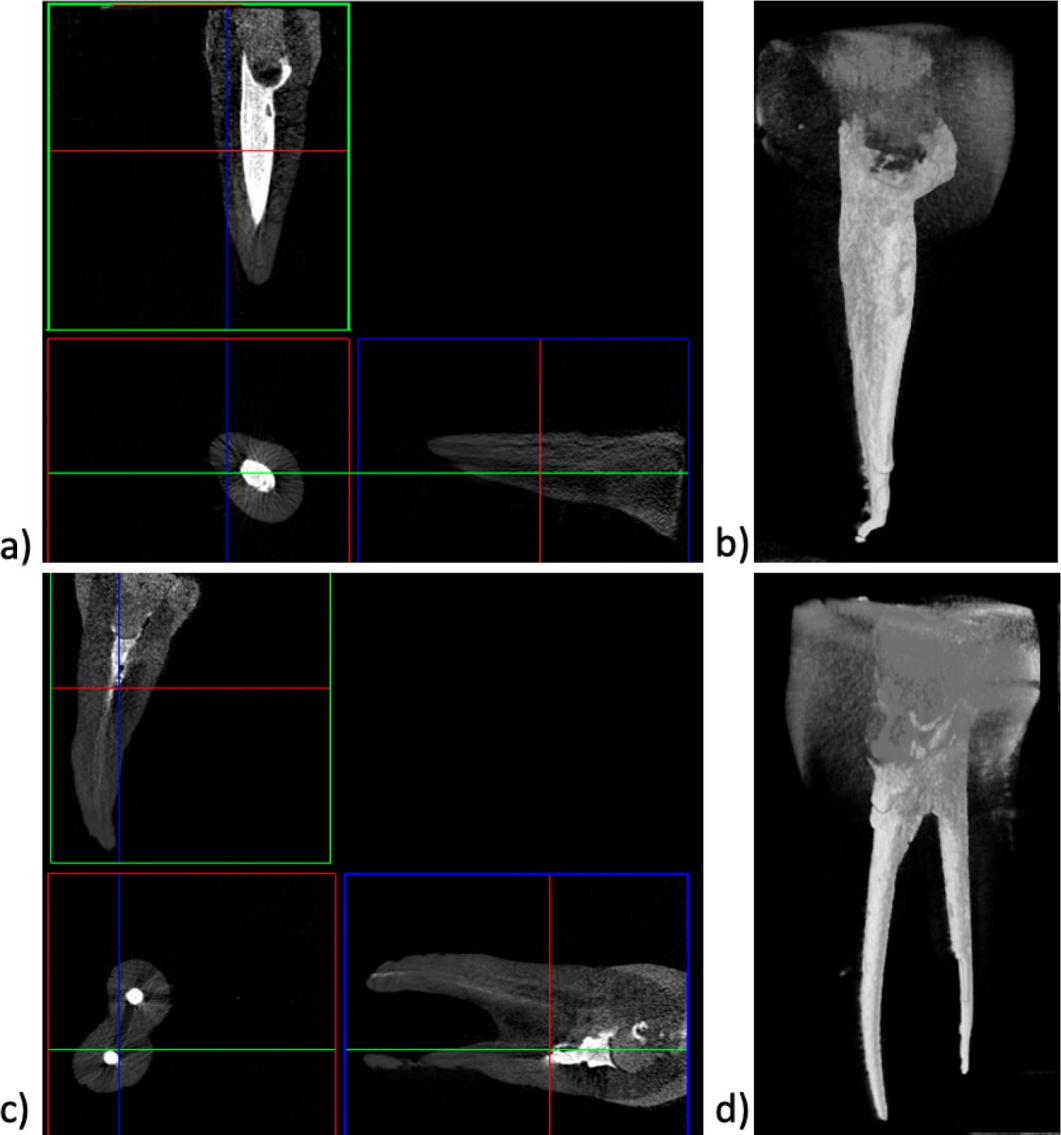
Images of single-rooted (**a**, **b**) and double-rooted (**c**, **d**) teeth restored with EverX Posterior, after compression testing—2D (**a**, **c**) and 3D (**b**, **d**); micro-CT

**Fig. 8:**
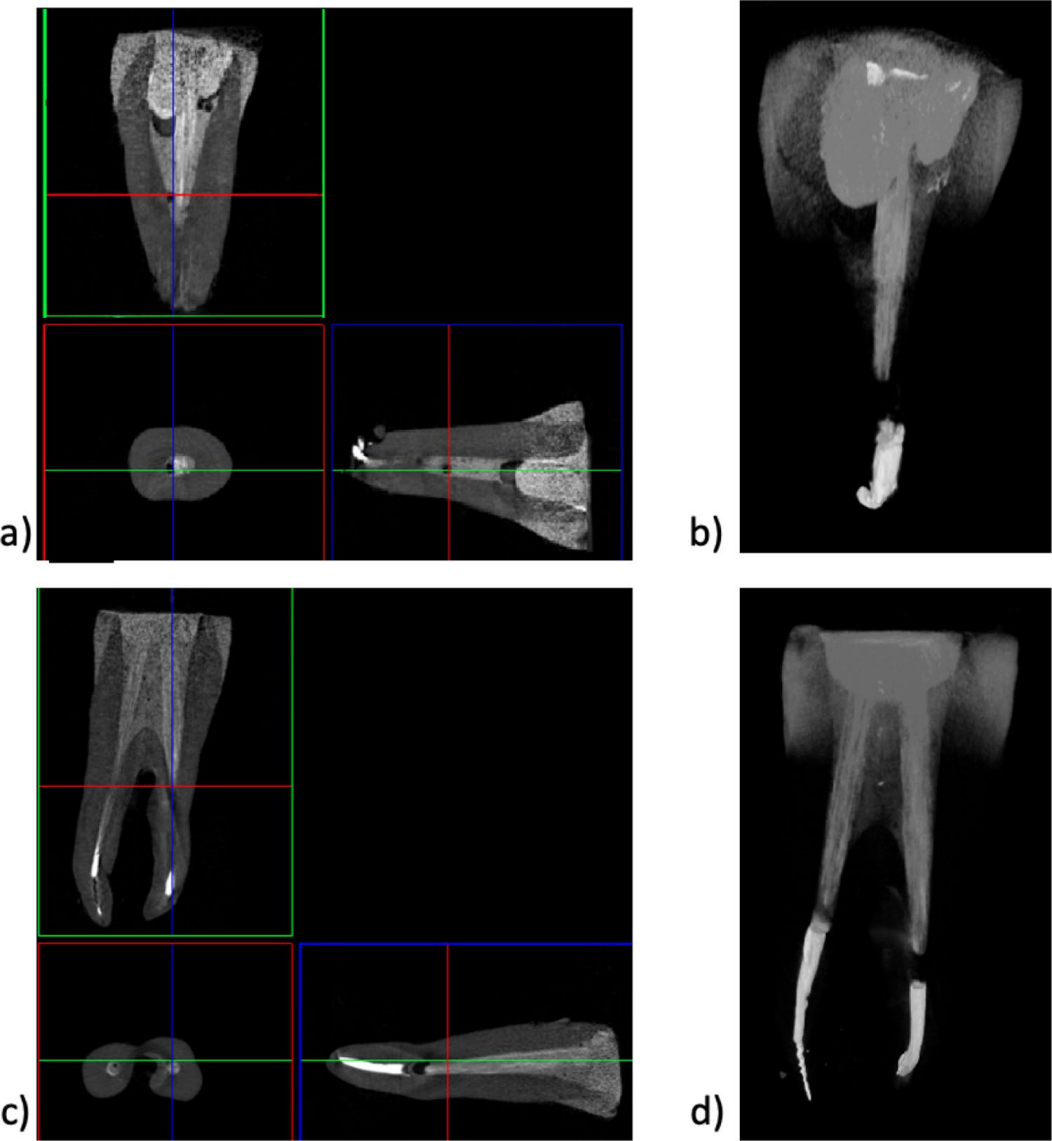
Images of single-rooted (**a**, **b**) and double-rooted (**c**, **d**) teeth restored with Hahnenkratt post, after compression testing—2D (**a**, **c**) and 3D (**b**, **d**); micro-CT

Figures 7 and 8 show the CT analysis results obtained after compressive strength testing for the teeth with EverX Posterior restorative material and continuous glass fibres posts Hahnenkratt, respectively.

An analysis of the X-ray images reveals no signs of degradation of the crown dentin, enamel and filling. The crown of the tooth is intact. One can observe no chipping of the dental structures and no damage between the filling and the tooth tissues (Figs. 7, 8).

#### Compressive Strength

Compressive strength results are given in Table 3.

**Table 3:** Compressive strength results

Materials	Max stress (MPa)	Max load (N)
Incisor teeth | Group 1	4.11 ± 0.12	292 ± 20.97
Premolar teeth | Group 1	4.73 ± 0.11	211 ± 16.98
Incisor teeth | Group 2	10.35 ± 0.25	498 ± 28.54
Premolar teeth | Group 2	9.77 ± 0.18	554 ± 37.44

Higher compressive strength values were obtained for the Group 2 teeth restored with continuous glass fibre posts Hahnenkratt rather than for the Group 1 teeth restored with short glass-fibre-reinforced composite EverX Posterior.

**Fig. 9:**
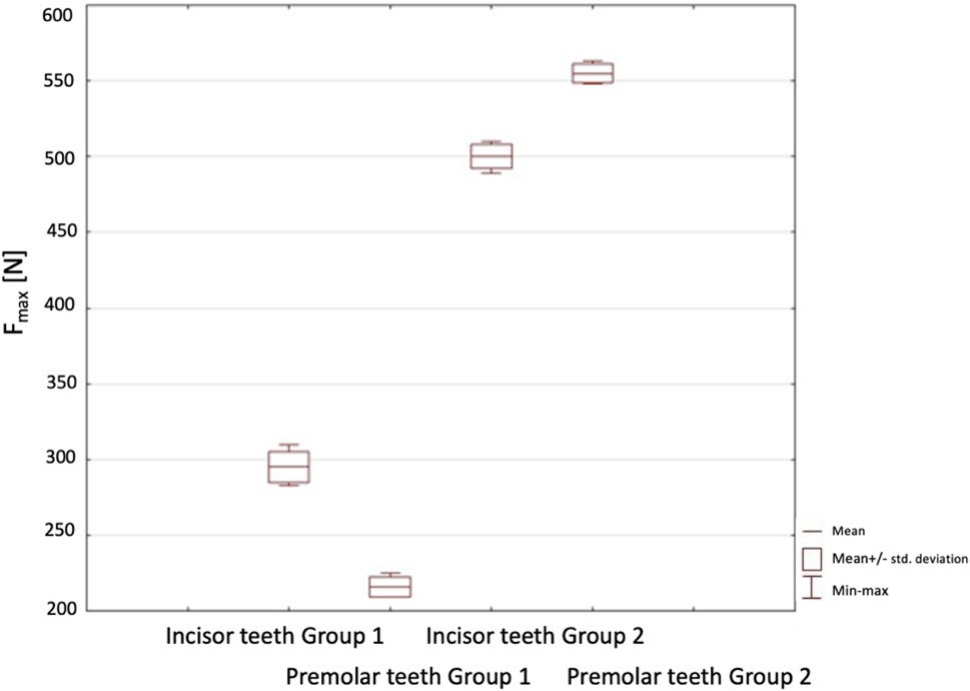
The box-and-whiskers plot of teeth strength for Group 1 vs. Group 2

Figure 9 shows box–whiskers graph of statistic of teeth between Group 1 and Group 2 and how the mean, min–max, and standard deviation for the analysed maximum force parameters are.

Based on the research, it was noted that the highest average maximum force was characterised by Premolar Hahnenkratt 555 N ± 6.4. Equally high results, about 10% lower, were obtained for Incisor Hahnenkratt, for which the average maximum force was 500.4 N ± 7.9. On the other hand, the lowest average maximum force, nearly 50% lower than that of Premolar Hahnekratt, was found in Premolar EverX Posterior 215.8 N ± 6.5. The use of Incisor EverX Posterior resulted in a nearly 37% increase in the average maximum force to 295 N ± 10.3 compared to Premolar EverX Posterior. Student’s *t *test showed that there are statistically significant differences, and *p* < 0.05. This means that, according to the analysis, there are significant differences between the analysed groups so at a significance level of *p* < 0.05 was for each Group.

A reinforcement with short undirected fibres produces isotropic properties, and the degree of strengthening primarily depends on the fibre volume. The use of unidirectional long fibre reinforcement provides anisotropic properties, with the highest strength observed parallel to the fibre direction. A combination of composite materials with different structures and properties is used to restore and fill endodontically treated teeth.

According to Randow and Glatz [20], pulp removal during a routine endodontic treatment results in a loss of positive feedback. The synergism of enamel, crown dentin, and root dentin gives rise to the formation of an integrated organ that can withstand high masticatory loads. The root dentin is an important structure integrating the dentition with the musculoskeletal support. The human root dentin has a higher bending strength and undergoes considerably higher inelastic deformation than the coronal dentin. Understanding the dentin's mechanical behaviour and complex relationships with the dentin structure provides insight into design strategies aimed at tooth function restoration and helps improve tooth restoration techniques to prevent catastrophic failures [20]. Post-endodontic treatment tooth restoration is a difficult procedure involving tissue reconstruction. The selection of an appropriate type of restorative material and accurate tooth restoration are of vital importance. The main reason for tooth extraction after root canal treatment is incorrect tooth reconstruction. An ideal post-endodontic tooth restoration should be able to transmit and distribute functional stresses while maintaining an adequate crown seal. The main factors affecting the tooth's resistance to fracture are the dimensions of a cavity and the properties of a restorative type of material. This can affect the relationship between filling and stress distribution and, thus, the mechanical response of the restored tooth.

The laboratory mechanical test demonstrated that the filling with short glass fibres had a considerably improved fracture toughness. The structure’s mechanical properties with continuous unidirectional fibres can yield better results than reinforcements with other fibres such as short and random fibres.

When a heavy load is applied to the tooth structure, the stresses acting on the continuous fibres are modified as the forces are absorbed and distributed by the tooth. This is possible as a result of a monoblock formed between the dentin and the restorative material. The fibres are designed to increase durability, stability and shear strength.

These fibres can control polymerisation shrinkage and marginal microleakage due to their orientation. A random fibre orientation plays a significant role in their mechanical properties. Stress transfer from the polymer matrix to the fibres is crucial for optimal polymer reinforcement. In this study, the damage to the teeth and fillings was adhesive, indicating that the interface is the weakest phase in tooth restoration.

The objective of this study was to evaluate the quality of two different reinforcements used for endodontically treated teeth (i.e. short fibres and posts). The first tested material was EverX Posterior, a composite reinforced with short glass fibres and the other material used for restoring the tooth after root canal treatment was Hahnenkratt’s glass-fibre-reinforced post.

The use of non-invasive microcomputed tomography made it possible to make a preliminary assessment of the internal structure of teeth without damaging them. In addition, CT made it possible to assess the quality of fillings and estimate the damage caused by compressive strength testing. The CT projections and images captured with a stereo microscope showed that applying a compressive force caused the enamel and dentin to crack. However, no damage was observed on the tooth tissue–filling interface.

The compression tests made it possible to investigate the behaviour of teeth with different fillings under oral cavity loading conditions. The compression test results demonstrated in this paper that the teeth restored with the use of a glass-fibre-reinforced post Hahnenkratt had higher compressive strength. Therefore, the restoration method studied in this article may minimise the risk of tooth root degradation after endodontic treatment.

As a results from Gaintantzopoulou et al., the combination of a glass-fibre-reinforced bulk fill liner EverX Posterior with a restorative showed a promising performance regarding the fracture mode, providing a better reinforcing effect that could serve as a less invasive and time-saving approach for the functional rehabilitation of preventing subsequent catastrophic failures [21].

Short glass-fibre-reinforced composite EverX Posterior in the restoration may improve the ability of the tooth-reinforcement complex to absorb occlusal loads [22, 23]. The positive effect of short glass-fibre-reinforced composite EverX Posterior posts in supporting composite restoration after endodontic treatment has been reported in several studies laboratory and clinical [21, 24–26].

It should also be taken into account that the tooth fillings were developed not in their natural environment, but under laboratory conditions. Following their extraction, until they were subjected to processing and testing, the teeth were stored in a solution imitating oral cavity conditions to prevent them from drying out.

The results of this study lead to the following conclusions:

1. The teeth restored with Hahnenkratt’s glass fibre posts showed higher compressive strength than those restored using the EverX Posterior material.
2. The tooth’s most weakened and sensitive point after endodontic treatment was the cervical area of the tooth. All cracks were parallel to the root canal.
